# Draft genomes, phylogenomic reconstruction and comparative genome analysis of three *Xenorhabdus* strains isolated from soil-dwelling nematodes in Kenya

**DOI:** 10.1099/acmi.0.000531.v4

**Published:** 2023-05-22

**Authors:** Ryan Musumba Awori, Charles N. Waturu, Sacha J. Pidot, Nelson O. Amugune, Helge B. Bode

**Affiliations:** ^1^​ Molecular Biotechnology, Department of Biosciences, Goethe University Frankfurt, 60438 Frankfurt am Main, Germany; ^2^​ Elakistos Biosciences, PO Box 19301-00100, Nairobi, Kenya; ^3^​ Horticulture Research Institute, Kenya Agricultural and Livestock Research Organisation, PO Box 220 Thika; ^4^​ Department of Microbiology and Immunology at the Doherty Institute, University of Melbourne, Melbourne, Australia; ^5^​ Department of Biology, University of Nairobi, PO Box 30197-00100, Nairobi, Kenya; ^6^​ Department of Natural Products in Organismic Interactions, Max Planck Institute for Terrestrial Microbiology, 35043 Marburg, Germany; ^7^​ Chemical Biology, Department of Chemistry, Phillips University Marburg, 35043 Marburg, Germany; ^8^​ Senckenberg Gesellschaft für Naturforschung, 60325 Frankfurt am Main, Germany

**Keywords:** prokaryotic pangenomics, *Steinernema *endosymbionts, species delineation, *Xenorhabdus *bacteria

## Abstract

As a proven source of potent and selective antimicrobials, *Xenorhabdus* bacteria are important to an age plagued with difficult-to-treat microbial infections. Yet, only 27 species have been described to date. In this study, a novel *Xenorhabdus* species was discovered through genomic studies on three isolates from Kenyan soils. Soils in Western Kenya were surveyed for steinernematids and *Steinernema* isolates VH1 and BG5 were recovered from red volcanic loam soils from cultivated land in Vihiga and clay soils from riverine land in Bungoma respectively. From the two nematode isolates, *Xenorhabdus* sp. BG5 and *Xenorhabdus* sp. VH1 were isolated. The genomes of these two, plus that of *X. griffiniae* XN45 – this was previously isolated from *Steinernema* sp. scarpo that also originated from Kenyan soils – were sequenced and assembled. Nascent genome assemblies of the three isolates were of good quality with over 70 % of their proteome having known functions. These three isolates formed the *X. griffiniae* clade in a phylogenomic reconstruction of the genus. Their species were delineated using three overall genome relatedness indices: an unnamed species of the genus, *Xenorhabdus* sp. BG5, *X. griffiniae* VH1 and *X. griffiniae* XN45. A pangenome analysis of this clade revealed that over 70 % of species-specific genes encoded unknown functions. Transposases were linked to genomic islands in *Xenorhabdus* sp. BG5. Thus, overall genome-related indices sufficiently delineated species of two new *Xenorhabdus* isolates from Kenya, both of which were closely related to *X. griffiniae *. The functions encoded by most species-specific genes in the *X. griffiniae* clade remain unknown.

## Data Summary

NCBI GenBank accession numbers of the three genome assemblies generated from this study are JACWFC000000000.1, JADEUF000000000.1 and JADEUG000000000.1. The metadata for soil samples collected in this study are listed in Table S2 (available in the online version of this article).

Accession numbers and strain names of publicly available genomes used in this study are listed in Table S3.

The supplementary workbook contains detailed raw data used for pangenome analyses. IS family transposases in the genome of strain BG5 have been deposited in the ISfinder Database under accession numbers ISXsp1, ISXsp2, ISXsp3, ISXsp4, ISXsp5, ISXsp6, ISXsp7, ISXsp8, ISXsp9, ISXsp10, ISXsp11, ISXsp12, ISXsp13, ISXsp14, ISXsp15, ISXsp16, ISXsp17, ISXsp18, ISXsp19 and ISXsp20.

Parts of the methods, results and discussion sections were previously reported in a doctoral thesis of the first author and are thus not considered a prior publication.

**Impact Statement**

*
Xenorhabdus* bacteria are important because they produce various antimicrobials. However, not many have been isolated from their natural habitat, which is the gut of soil-dwelling *Steinernema* nematodes. Two of these bacteria were thus isolated from nematodes of soils in Western Kenya. Their genome sequences were determined and used in genomic analyses, which revealed novel species. These genomes can be used to show the location of genes encoding antimicrobial production, thus making it easier for future isolation of antimicrobials from these new bacterial strains.

## Introduction

Bacteria of the genus *Xenorhabdus* naturally produce specialized metabolites such as non-ribosomal peptides that have antiprotozoal, antifungal and antibacterial activities [1]. Yet, despite each *Xenorhabdus* species [2] – and sometimes even strain [3]– encoding a unique antimicrobial production profile, only 27 [4–13] *Xenorhabdus* bacteria have been isolated from their *Steinernema* nematode hosts and described as novel species. Thus, the aim of this study was to discover new *Xenorhabdus* species and strains.

Bacteria of the genus *Xenorhabdus* are naturally found in soil biota, specifically as autochthonous endosymbionts of insect-killing *Steinernema* nematodes. Once a steinernematid enters an insect prey via natural openings such as spiracles, it migrates to the haemocoel and defecates [14] its *Xenorhabdus* endosymbionts, which then secrete specialized metabolites such as insecticidal toxins [15] that result in quick death of the insect. Secreted antimicrobials function to deter soil microbial competitors from the nutrient-rich cadaver. Nematodes then utilize this nutrient-filled, cadaver enclosure to reproduce exponentially. Depletion of nutrients halts the reproductive cycle and triggers nematode bacterium re-association. This is succeeded by pre-infective juvenile steinernematids emigrating from the cadaver to the soil, where they lie in wait for insect prey. Thus, to isolate a *Xenorhabdus* bacterium one needs to first isolate its infective juvenile steinernematid host from soil, using a combination of insect larvae as bait [16] and White traps [17].

For species such as *Xenorhabdus khoisanae* (Table S1), *X. bovienii *, *X. kozodoii *, *X. poinarii* and *X. hominckii,* we see one *Xenorhabdus* species as the natural symbiont of numerous *Steinernema* species [18]. However, the reverse, one *Steinernema* species that naturally hosts, with equal fitness, two different *Xenorhabdus* species, has yet to be discovered. Thus, there is a high possibility of identifying new *Xenorhabdus* species from the over 50 described *Steinernema* species whose symbionts remain uncharacterized [19].

This research gap between steinernematid isolation and *Xenorhabdus* endosymbiont identification is also seen in Sub-Saharan Africa, where numerous steinernematids have been isolated (see Table S1 for a full list of species and location). In Kenya, apart from *X. hominickii* from *Steinernema karii* [13], and *X. griffiniae* XN45 that we isolated from *Steinernema* sp. scarpo [20], endosymbionts have yet to be isolated and described from strains that were isolated from Central and Coastal Regions including *Steinernema* sp. UH3 [21] and UH13 [22]. No steinernematid isolates have been documented from the Western region of Kenya.

Genome assemblies of >50× coverage of new isolates are not only required for the description of novel species/emendation of prokaryotic taxa [23] but also enable accurate species delineation via overall genome-related indices (OGRIs) such as orthologous average nucleotide identity (orthoANI) [24] and digital DNA–DNA hybridization (dDDH) [25], and phylogenomic reconstructions based on genome-genome distances [26]. Furthermore, comparative genome analyses of closely related strains are useful for the identification of not only genomic islands and mobile genetic elements but also the core and dispensable genes of a specific monophyletic group [27, 28].

In this study two *Xenorhabdus* strains, VH1 and BG5, were isolated from soil biota from Western Kenya. Their genomes, and that of *X. griffiniae* XN45 that we previously isolated from *Steinernema* sp. scarpo from Kenya, were sequenced and assembled. These three genomes were used for downstream species delineation and emendation, and comparative genome analyses.

## Methods

### Collection of field soil samples

Fieldwork was carried out from 16 October 2018 to 4 November 2018 in the Western and Rift Valley Regions of Kenya. No access permits were required as per the exceptions of section 3(d) of the Environmental Management and Coordination (Conservation of Biological Diversity and Resources, Access to Genetic Resources and Benefit Sharing) Regulations 2006 of the Environmental Management and Coordination Act, 1999 of the Laws of Kenya. Ten localities were selected for the collection of soil samples: Nandi Hills, Tinderet, Fort Tenan, Kakamega, Gisambai, Vihiga, Kisumu, Bungoma, Kaimosi and Mt. Elgon. Within each locality, collection points were selected from cultivated lands, fallow lands, forests, crop edges, shorelines, swamps and riverine areas. This resulted in a total of 76 soil collection points. To collect a soil sample, vegetation was first cleared from the topsoil. Then using a digging fork, soil was excavated to a depth of not more than 60 cm. Using a collection spade, the soil was scooped into a measuring cup to an amount of ca. 500 g. Twigs, branches and stones were removed before the soil sample was placed in labelled cotton bags. Dug-out soil was returned to the hole and soil samples were then transported at room temperature to the laboratories. Geographical coordinates, altitude and descriptions of soil collection points are provided in Table S2.

### Isolation of nematodes from soils samples

To isolate entomopathogenic nematodes (EPNs) from soils, a soil sample was first spread out on a tray and crumbled. The soil was then redistributed into transparent polyethylene terephthalate (PET) plastic containers of 20 cm in diameter and 5 cm in depth. To bait EPNs from the soil, *Galleria mellonella* larvae were first obtained from a laboratory insect culture of KALRO-Horticulture Research Institute, where they had been reared as previously described [29]. From this culture, any healthy larvae were selected. Two/three larvae were then buried in the soil, in a hole of ca. 1 cm diameter and 5 cm depth. In total, about 15 *G*. *mellonella* larvae buried in five holes per container were used as bait. The container lids were replaced and set-ups were maintained at room temperature. After a maximum of 7 days, containers were checked. Dead larvae were assessed for the following characteristics that typify an EPN infection: limp cadaver, tan or red in colour, and minimal smell of putrefaction. Samples BG5 and VH1 had dead cadavers that were either light red or tan in colour. Sample BG5 was clay soil collected from fallow riverine land. Sample VH1 was collected from land cultivated with cabbages. To isolate putative EPNs from these cadavers, a modified White trap [17] was used. Briefly, clean PET containers of 20 cm diameter and 5 cm depth were filled with distilled water to a depth of ca. 4 mm. A clean Petri dish was placed upside down into the container such that the Petri dish surface was raised from the bottom of the PET container. Clean white cotton cloths of the same size as the Petri dish were placed on this raised surface. Selected cadavers were placed onto the cotton cloths. To allow putative EPNs to emigrate from the cadavers to the water, a part of the cloth was dipped in the distilled water. PET containers were covered and kept for 7 days. The distilled water was observed daily under a dissecting microscope for the presence of white motile, ca. 1 mm long nematodes. For positive samples, contaminants such as cadaver tissue debris were separated from nematodes by a series of sedimentation and decanting using clean distilled water. Nematodes were stored in contamination-free distilled water – to a depth of not more than 0.4 cm – in clear plastic containers in the dark. Stored EPN nematode cultures were named after their soil collection point.

### Isolation of bacteria from nematodes

The indirect isolation of *Xenorhabdus* bacteria from haemolymph was based on a previously described method [30] with modifications [20]. First, nematode isolates from collection points BG5 and VH1 were selected. Cadavers with which BG5 and VH1 nematodes were baited were surface-sterilized and dissected under aseptic conditions. A light-yellow, viscous, heterogenous fluid was aseptically obtained and streaked onto nutrient agar supplemented with 0.0025 % (w/v) bromothymol blue and 0.004 % (w/v) 2,3,5 triphenyl tetrazolium chloride (NBTA). This was incubated at 30 °C for 96 h. Only colonies that had the following observed morphologies were selected for further pure culture techniques: blue/yellow pigmentation, irregular margins, umbonate shape and visible swarming patterns. On these pure cultures, a catalase test was performed, and the absence of bubble production indicated a catalase-negative isolate, and these were presumptively identified as *Xenorhabdus* species. They were named *Xenorhabdus* sp. strains BG5 and VH1.

### Genome sequencing and assembly

Previously, we isolated *X. griffiniae* XN45 from *Steinernema* sp. scarpo, which was originally isolated from Muran’ga District in Kenya [20]. Thus, in addition to *Xenorhabdus* sp. strains VH1 and BG5, this strain was selected for genome sequencing and assembly. DNA from strain XN45 was extracted with FastDNA Spin Kit for Soil (Mp Bio) to yield a concentration of 20 ng µl^–1^ and UV absorbance ratio at 260_nm_/280_nm_ (A260/280) >1.8. From this, only 0.1 ng of DNA was used to prepare a library using a Nextera XT kit (Illumina). Sequencing was done by CeGaT GmBH on a NovaSeq 6000 platform with the following parameters: short insert paired-end reads of 100 bp and targeted coverage of 100×. Output data were raw sequence reads in fastq.gz format (2.902 GB), which had Illumina standard Phred scores (offset +33) and adapter sequences already removed. In terms of quality, 91.32 % of reads had a Q30 value. Genome assembly was done with Spades 3.10.1 [31] with thresholds for minimum contig length and coverage set at 1000 bp and 5× respectively.

For VH1 and BG5, DNA was isolated with Gentra Puregene DNA extraction kit (Qiagen) to yield samples of 1 µg µl^–1^ concentration and A260/280 ratios of >1.8. At the Doherty Institute, University of Melbourne, Australia, DNA libraries were created using a Nextera XT DNA preparation kit (Illumina), and whole genome sequencing was performed on a NextSeq platform (Illumina) with paired-end reads of 150 bp and targeted coverage of >50×. Genome assembly was done with Spades 3.10.1 [31]. For assembled genomes of strains VH1, BG5 and *X. griffiniae* XN45 from this study and *X. griffiniae* strain BMMCB (doi: 10.1128/genomeA.00785–15) from Mothupi *et al*. [32], characteristics such as completeness, contamination, N50, L50, length and GC content were determined using the comprehensive genome analysis tool of the PATRIC platform [33].

### Phylogenomic reconstruction and calculation of ANI values

For phylogenomic reconstruction of the genus *Xenorhabdus *, 27 fasta files (Table S3) were used as input data for a whole genome-based taxonomic analysis on the Type strain genome server platform (TYGS) [26]. On these, the MASH algorithm [34] was used to quickly calculate intergenomic relatedness and determine the strains with the smallest distances. All pairwise comparisons and inference of intergenomic distances among the set of genomes were conducted using the genome BLAST distance phylogeny (GBDP) 'trimming' algorithm and distance formula *d5* [35]. One hundred distance replicates were calculated for each. The genome–genome distance calculator (GGDC) 2.1 formula was used to calculate dDDH values and confidence intervals [35]. Confidence intervals are given in workbook S1. Intergenomic distances were then used to infer a balanced minimum evolution tree with branch support via FASTME 2.1.4 including SPR post-processing [36]. Branch support was inferred from 100 pseudo-bootstrap replicates for each. Rooting of trees was done at the midpoint whereas visualization and graphics editing were done with iTOL [37] and Inkscape [38] respectively.

Using the same workflow, a phylogenomic reconstruction with only strains VH1, BG5, BMMCB and XN45 was made. Minimum thresholds for two strains to be classified as one species and sub-species were 70 % and 79 % dDDH respectively [26]. To calculate ANI values among species most closely related to strains XN45, VH1 and BG5, the orthoANI algorithm was used within the OAT software package, which was also used to obtain genome–genome distance (GGD) 2.1 values [24].

### Creation of pangenomes

To determine whether the genus *Xenorhabdus* had an open pangenome, genomes of *Xenorhabdus* species were first used to construct a pangenome of the genus using the anvio v7.1 pangenome workflow [28, 39] with the following parameters: use ncbi-blast, MCL inflation=10, minbit=1, exclude-partial-gene-calls. A pangenome of strains VH1, BG5, XN45 and BMMCB only was also created using the same workflow and parameters. The strain names, accession numbers and total number of gene calls of each genome used are listed in Table S3. The mean α value was determined using the P-GAP platform running on the Panweb server [40]. Briefly, RAST-k [41] annotated genomes were used as data input on the Panweb server and the following parameters were selected for clustering genes into one gene cluster: minimum 80 % nucleotide similarity, and minimum 80 % coverage with gene family algorithm.

### Estimation of the effect of draft genomes on the determination of the core genome

To estimate how the use of draft genomes affects the determination of the core genome, two additional pangenomes were created. The first contained six genomes, each of which was composed of fewer than two contigs: *X. bovienii* CS-03 (NZ_FO818637), *X. hominickii* ANU (NZ_CP016176), *X. cabanillasii* DSMZ 19705 (NZ_QTUB01000001), *X. poinarii* G6 (FO704551), *X. nematophila* AN6/1 (FN667742) and *X. szentirmaii* US123(NIUA01000001). The second contained draft genomes of similar species: *X. bovienii* T228 (JANAIF000000000.1), *X. hominickii* DSM 17903 (NJAI00000000.1), *X. cabanillasii* JM26 (NJGH00000000.1), *X. poinarii* SK (JADLIG000000000.1) and *X. nematophila* C2-3 (JRJV00000000.1). Pangenomes were created via the aforementioned anvio workflow and the sizes of their resultant core genomes were compared.

### Analysis of gain and loss of gene clusters in the *X. griffiniae* clade

Using the gene clusters (GCs) of the VH1-BG5-XN45-BMMCB pangenome, a matrix of the presence and absence of GCs among the four strains was created (workbook S1). This matrix and the GBDP phylogeny of the four strains were then used as input data for gene gain and loss analysis in the COUNT program (downloaded 17 January 2023) using Wagner parsimony (penalty=1) [42].

### Characterization of core, accessory, species and strain-specific genes

By using the ‘search’ and ‘bin’ functions of the anvi´o-interactive program, GCs that were present in all genomes under analysis as single copies were obtained and binned as single-copy core GCs (SCGs). Other binned GCs were: strain BG5 specific, strain BMMCB specific, strain XN45 specific, strain VH1 specific, XN45-VH1 accessory/* X. griffiniae* species specific, XN45-VH1-BG5 accessory, XN45-VH1-BMMCB accessory and BMMCB-BG5 accessory. Using the anvi-get-sequences-for-gene-clusters program with the ‘report DNA sequences’ and ‘concatenate’ flags, sequences for the single-copy GCs of each of the bins were obtained. For those consisting of GCs that constituted genes from multiple genomes, sequences from a single genome were used to represent the GC. These sequences were annotated in PROKKA [43] to elucidate the functions encoded by predicted genes.

### Clustering of gene clusters into functional groups

The functions of GCs were determined by manually querying the UniProt Knowledgebase [44] with each annotated gene symbol. Then, GC functions were assigned based on the described biological process the gene most clearly contributed to. This was supplemented by querying GCs with assigned cluster of orthologous groups (COG) functions against the respective database [45]. To aid the identification of genes that encode the biosynthesis of specialized metabolites, nucleotide sequences for each bin were annotated in antiSMASH [46]. Then, GC-function lists were compiled for each bin and manually curated. GCs were grouped according to similarity of function and visually represented in column graphs.

### Elucidation of genomic islands in strain BG5

To highlight putative genomic islands flanked by transposase genes, an annotated record of the BG5 genome was concatenated and used as the reference genome in BRIG [47] and compared to genomes of VH1 and XN45 by the blast algorithm [48] utilizing an NCBI-blast 2.4.0+ bin library. Selected rings to be visualized were for BG5 genome guanine-cytosine (G+C) content (ring 1) and skew (ring 2), VH1 genome (ring 4), XN45 genome (ring 5) and loci of coding DNA sequences (CDS) annotated as transposases on the BG5 genome (ring 6). Output visualizations were obtained as .svg files and enhanced in Inkscape [38].

## Results

### Strains VH1, BG5, XN45 and BMMCB form a clade

Two putative *Steinernema* isolates, VH1 and BG5, were isolated from soils in Western Kenya. VH1 was isolated from red volcanic loam soils on cabbage cultivated land at a point with coordinates 0.06293, 34.72903 and altitude 1624 m, in Vihiga. BG5 was isolated from clay soils on riverine land at a crop edge at a point with coordinates 0.48044, 34.40836 and altitude 1239 m, in Bungoma. From these two, *Xenorhabdus* sp. strains VH1 and BG5 were respectively isolated. Soils from the Rift Valley Regions sampled did not yield any steinernematids. Previously, we isolated *X. griffiniae* XN45 from *Steinernema* sp. scarpo, which originated from soils in Muran’ga County, Kenya [20]. *Xenorhabdus* sp. strain BMMCB, which was designated as an *X. griffiniae* species, was previously isolated from *Steinernema* sp. BMMCB [32], whose natural habitat was red volcanic sandy-loam soils in Brits, North West Province, South Africa [49].

Thus, to investigate the phylogenomic relationships between these four strains, the quality of their genome assemblies was first determined. The draft genomes of XN45, VH1, BG5 and BMMCB were complete and consistent, with low contamination (Table 1), and of coverage >50×. They were thus of sufficient quality for species delineation via overall genome relatedness indices [23]. For the nascent BG5, XN45 and VH1 assemblies, 74.5, 72.2 and 71.5 % of proteins encoded in their respective genomes had known functions (Table 1), which were slightly below the 80 % average for genomes of *Gammaproteobacteria* [45].

**Table 1.** Quality and characteristics of genome assemblies Genomes of BG5, XN45 and VH1 were assembled in this study. BMMCB was obtained from NCBI GenBank (LDNM00000000.1). Annotation statistics were determined via the PATRIC platform.

	* Xenorhabdus* sp*.* strain BG5	* X. griffiniae* XN45	* Xenorhabdus* sp*.* strain VH1	* Xenorhabdus* sp*.* strain BMMCB
Contigs	129	381	273	231
Guanine–cytosine content (%)	43.80	43.57	43.65	44.68
Contig L50	12	29	43	21
Contig N50 (bp)	102 633	45 29	29 298	57 901
Genome length (bp)	3 933 551	4 215 754	4 224 998	4 183 760
Fine consistency (%)	96.5	95.9	95.9	95.7
Coarse consistency (%)	97.0	96.7	96.7	96.7
Contamination (%)	0.7	0	0	0
Completeness (%)	100	100	100	100
CDS	3827	4232	4160	4318
Repeat regions	127	66	69	70
Hypothetical proteins	973	1175	1185	1193
Proteins with functional assignments	2854	3057	2975	3125

Bp, base pairs; CDS, coding DNA sequences; Contig L50, the minimum number of contigs that contain 50% of the assembly; Contig N50, the shortest contig among that minimum number of contigs, which contain 50% of the assembly.

**Fig. 1.**
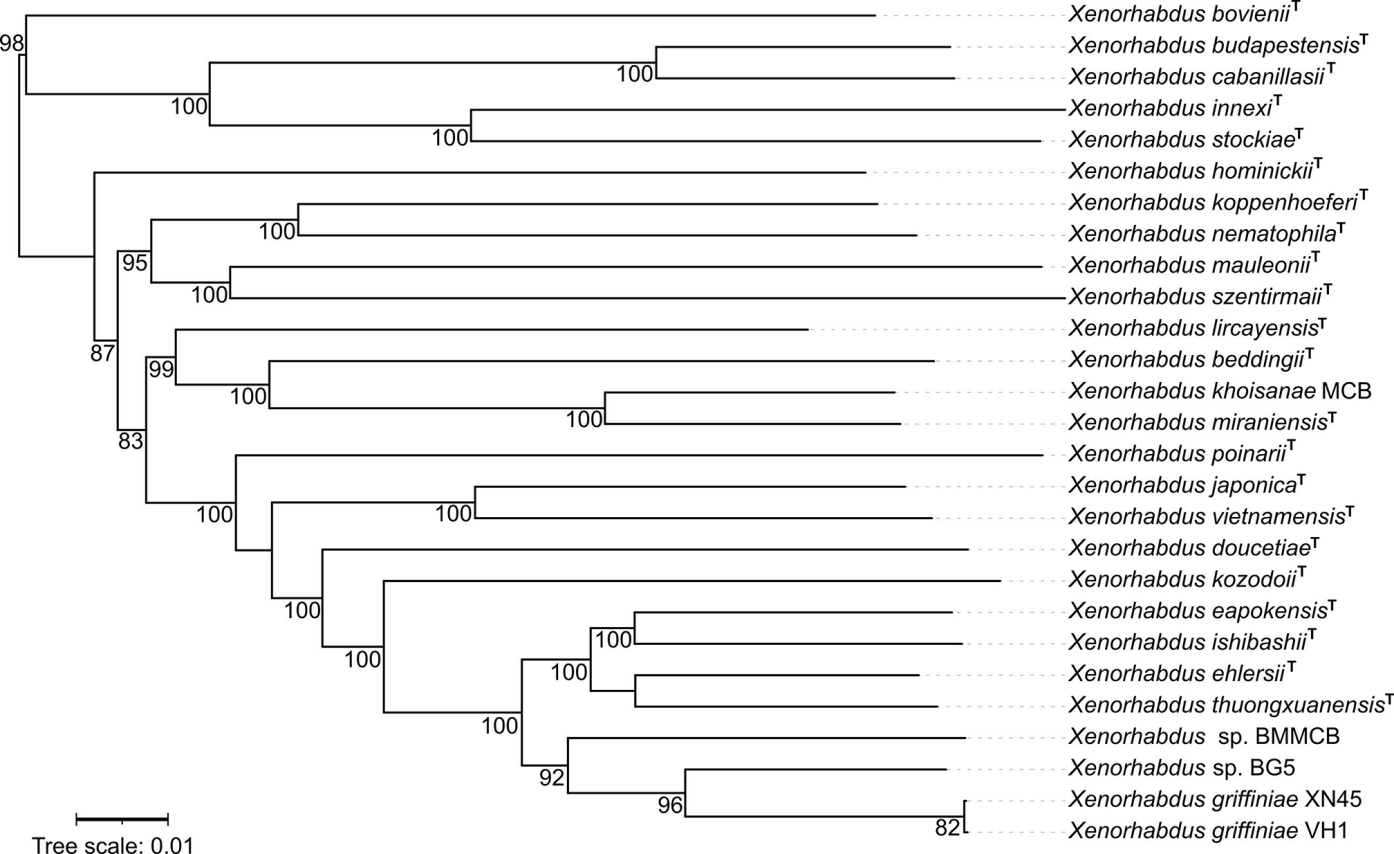
Phylogenomic reconstruction of *Xenorhabdus* species using genome blast distance phylogeny approach (GBDP) distances calculated from genome sequences using the *d_5_* distance formula. Genome sequences of *Xenorhabdus* sp. strains VH1 and BG5 and *X. griffiniae* XN45 were obtained in this study. *Xenorhabdus* sp. strain VH1 and *X. griffiniae* XN45 clustered together. *Xenorhabdus* sp. strain BMMCB was previously classified as representing an *X. griffiniae* species. However, it did not cluster with *X. griffiniae* XN45. *Xenorhabdus* sp. strain BG5 did not form a clade with any extant species. GBDP pseudo-bootstrap values of above 40 % are shown. The scale bar represents substitutions per site.

**Fig. 2.**
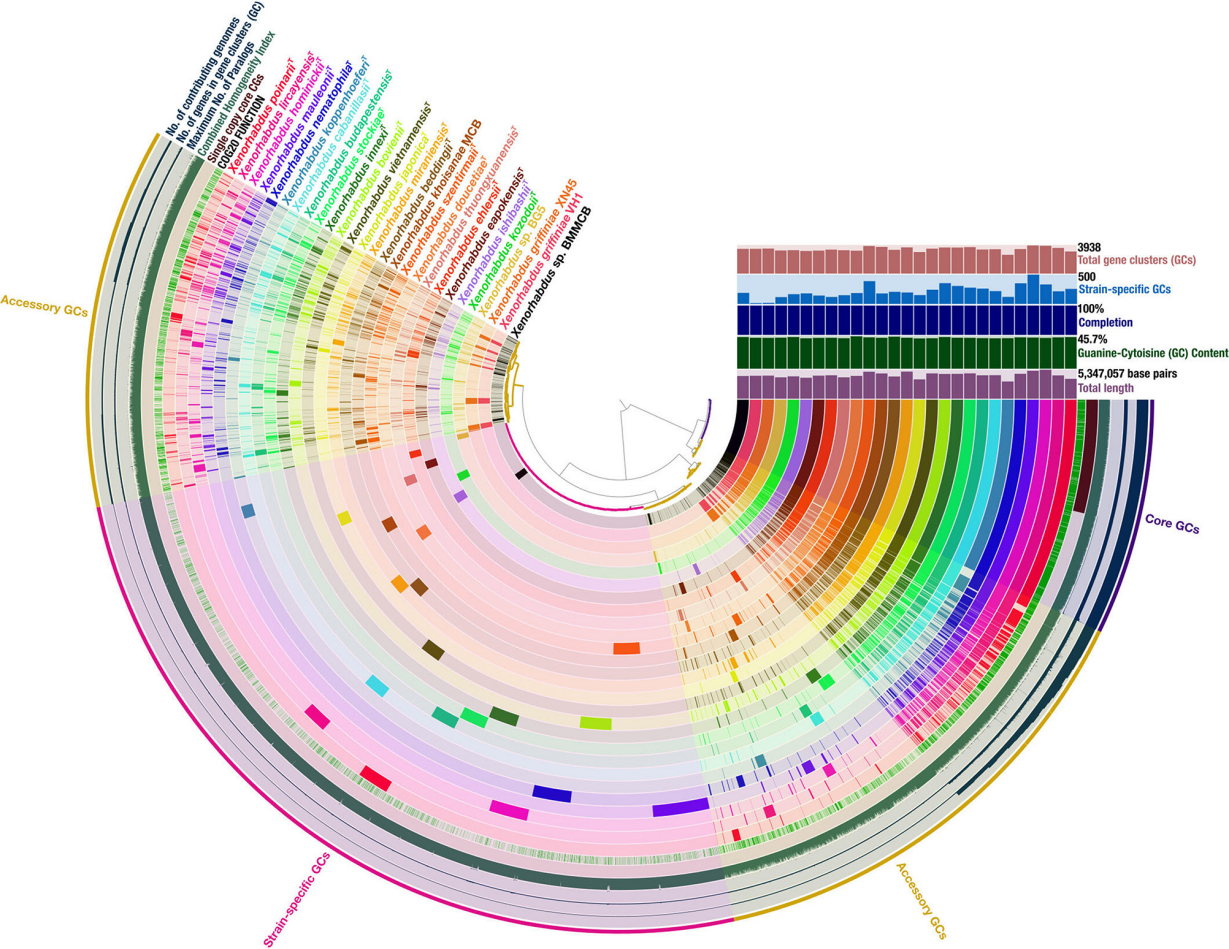
Graphical representation of the pangenome of 26 species of the genus *Xenorhabdus *. The largest genome was 5 347 057 bp and the highest guanine–cytosine content was 45.7 %. The pangenome was composed of a total of 13 469 gene clusters (GCs). Core GCs, those found in all 27 genomes, numbered 1654 in total. Accessory GCs, those found in two to 26 genomes, numbered 5992 in total. Strain-specific GCs, those found in one genome only, numbered 5820 GCs in total.

Strains VH1, BG5, BMMCB and XN45 formed an exclusive clade, as demonstrated in a phylogenomic reconstruction of 24/27 described species of the genus (Fig. 1). They were more closely related to each other than to other species, and this is demonstrated by the most closely related strains to VH1, BG5, XN45 and BMMCB being XN45, XN45, VH1 and BG5 (Fig. 2) at genome–genome distances (GGD) of 0.00, 0.059, 0.00 and 0.08 respectively (Table 2).

**Table 2.** Orthologous average nucleotide identity (orthoANI), genome-to-genome distance (GGD) and digital DNA–DNA hybridization (%) (dDDH) values for type species most closely related to *Xenorhabdus* sp. strains VH1 and BG5, *X. griffiniae* XN45 and *Xenorhabdus* sp. strain BMMCB OrthoANI values are in the top half of the matrix (top triangle), GGD in parentheses, and values for dDDH are in the bottom half of the matrix (bottom triangle). Values that are within the threshold for two strains to be classified as one species are shaded in grey. The thresholds for conspecific strains are orthoANI values above 95.1 %, dDDH values above 70 % and GGD values below 0.0361. Type strains are: DSM 2270*, X. ishibashii*; DL20*, X. eapokensis*; DSM 16337*, X. ehlersii*; 30TX1*, X. thuongxuanensis*.

	DSM 22 670	XN45	DL20	DSM 16 337	VH1	BG5	30T×1	BM MCB
BM MCB	89.68 (0.103)	91.41 (0.088)	90.03 (0.010)	91.95 (0.082)	91.42 (0.088)	92.25 (0.080)	90.56 (0.094)	–
30T×1	92.55 (0.076)	91.02 (0.091)	93.42 (0.067)	93.76 (0.064)	91.01 (0.091)	91.72 (0.085)	–	49.6
BG5	90.44 (0.097)	94.24 (0.059)	90.94 (0.092)	93.08 (0.071)	94.22 (0.059)	–	57.3	57.1
VH1	89.80 (0.102)	99.99 (0.000)	90.32 (0.097)	91.95 (0.081)	–	67.4	51.1	50.5
DSM 16 337	92.11 (0.080)	92.05 (0.081)	92.55 (0.077)	–	59.9	66.7	53.50	56.4
DL20	93.23 (0.069)	90.34 (0.097)	–	47.90	51.6	57.5	51.90	50.9
XN45	89.92 (0.102)	–	51.7	59.9	99.9	67.3	51.1	50.4
DSM 22 670	–	50.3	51.2	46.4	50.3	55.0	48.3	48.9

Strains XN45 and VH1 were conspecific, as demonstrated by their GGD, dDDH and ANI values of 0.000, 99.9 % and 99.9 % respectively– these were all within the conspecific thresholds of <0.0361 for GGD, >70 % for dDDH and >95.1 % for orthoANI [24, 26, 35]. XN45 and BG5 were most closely related to each other but were not conspecific as their GGD, dDDH and ANI values of 0.059, 67.3 % and 94.24 % respectively were all outside conspecific thresholds. BG5 and BMMCB were most closely related to each other but were not conspecific, as seen from their GGD, dDDH and ANI values of 0.08, 57.1 % and 92.25 % respectively.

BMMCB and XN45 were both described as *X. griffiniae* species [20, 32]. We previously demonstrated XN45 and *X. griffiniae* ID10^T^ as conspecific based on percentage nucleotide similarities for their 16S rRNA, *recA* and *serC* genes as 99.595, 98.571 and 97.686 % respectively. These were above the same species thresholds of 98.65, 97 and 97 % respectively [11, 50]. Conversely, strains BMMCB and XN45 were not conspecific, as demonstrated by their respective percentage nucleotide similarities values of 98.545, 93.67 and 92.066 % for 16S rRNA, *recA* and *serC* genes respectively [20]. Indeed, BMMCB was not an *X. griffiniae* species as its GGD, dDDH and ANI values with *X. griffiniae* XN45 were 0.08, 50.4 % and 91.41 % respectively – these were all outside conspecific thresholds. This was corroborated by a difference of 1.11 % in G+C content between the two genomes (Table 1), which was above the 1 % same species threshold [51]. Taken together, these results demonstrated that these four strains represented three species: *X. griffiniae* XN45, *X. griffiniae* VH1, and the two undescribed species *Xenorhabdus* sp. BG5, and *Xenorhabdus* sp. BMMCB.

### The genus *Xenorhabdus* has an open pangenome

We hypothesized that the genus *Xenorhabdus* had a pangenome that included many strain-specific genes, due to the numerous *Xenorhabdus* strains and their respective genomes, which have not yet been isolated from under-investigated *Steinernema* species [19]. This would make it an open pangenome. The pangenome is the pool of genes from which all the taxon genomes are constituted. The core genes are those found in all genomes, accessory genes are those found in two or more genomes, and strain-specific genes are those found in one genome only [27].

The *Xenorhabdus* pangenome was composed of 27 genomes (from 24/27 described species) each of which had between 2771 (* X. koppenhoeferi *) and 4990 (* X. hominickii *) genes. It contained 101 832 genes, after the exclusion of 3668 partial gene calls from the analysis (Table S2). The pangenome contained a total of 13 469 GCs (Fig. 2). The core genome had 1654 GCs (12.3 %). However, this likely to be an underestimate since draft genomes were mostly used in the analysis, and the use of draft as opposed to complete genomes reduced the core genome size by 5 %, in our comparison of pangenomes of similar species (Fig. S3). The accessory genome had 5992 GCs (44.5 %) and the strain-specific genome had 5820 GCs (43 %). In total, 6834 GCs (50.7 %) encoded known functions as per the Clusters of Orthologous Genes (COG) database. The highest percentage of these were in the core genome whereas the strain-specific genome had the lowest. *X. mauleonii* had the largest number of strain-specific genes (500) whereas *X. ehlersii* had the fewest (118). *X. griffiniae* VH1 and XN45 had remarkably few strain-specific GCs, 13 and 17 respectively, as they were the only two strains from the same species (Fig. 2). Strain-specific genes from newly added genomes increase the pangenome, and the rate of new strain-specific genes per newly added genome decreases to zero. If this rate of decrease is high, the result is a closed pangenome [27] whose size does not change significantly with the addition of new genomes. Conversely, open pangenomes have a slow rate of decreasing number of new strain-specific genes per newly added genome. Moreover, they are typified by a small percentage of core genes [52]. Relatedly, the mean α exponent of Heaps' Law is used to estimate this rate of decrease [53], and pangenomes with values >1 are defined as closed whereas those <1 are defined as open [27]. Thus, *Xenorhabdus* has an open pangenome, as demonstrated by its mean α exponent of Heaps' Law of 0.2752 and a core genome of 12.2 % (1654/13 469). This corroborated previous global comparative genome analyses of the genus *Xenorhabdus* [2, 28, 54].

### Most species-specific genes in a pangenome of the *X. griffiniae* clade encode unknown proteins

**Fig. 3.**
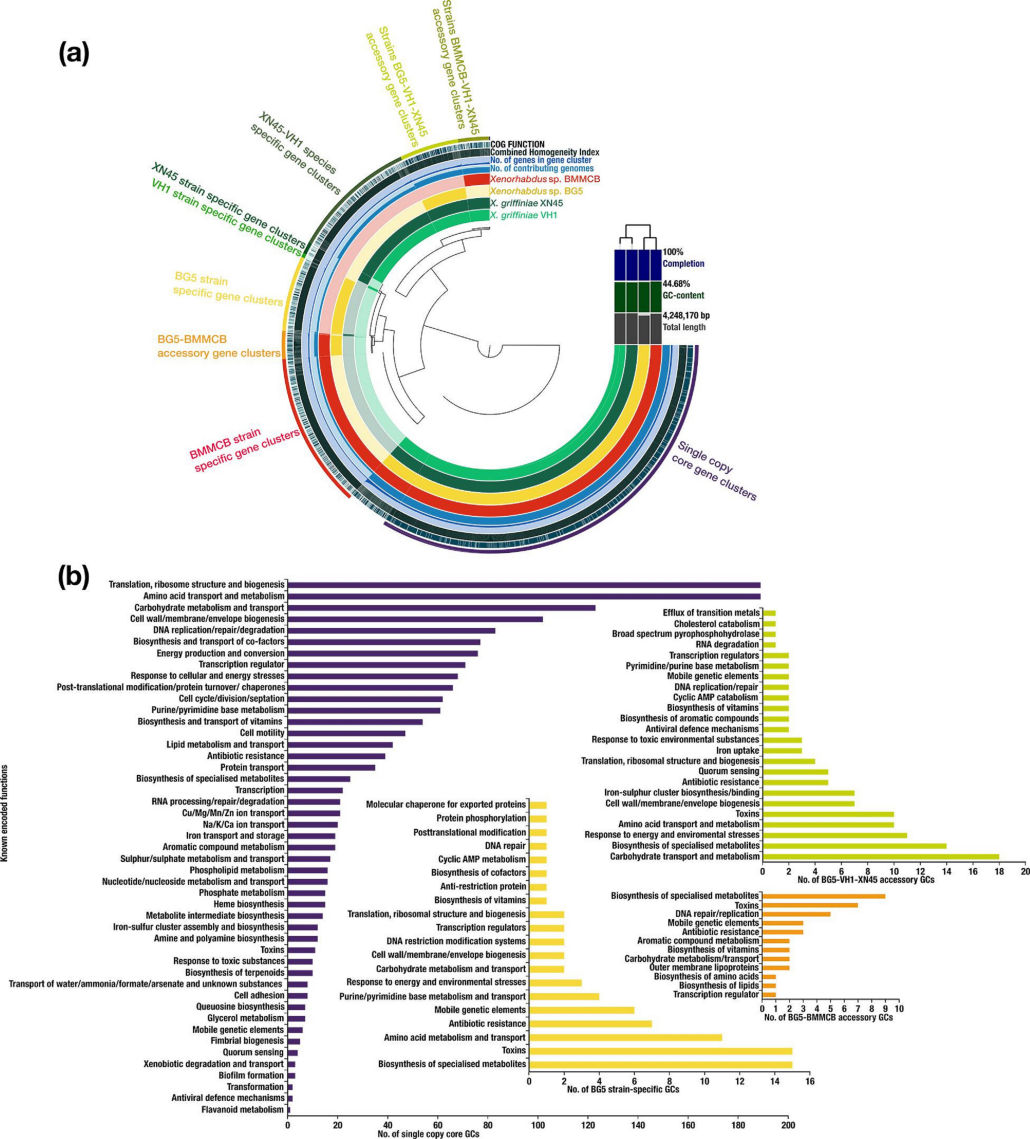
(a) Graphical representation of a pangenome of a monophyletic group of three *Xenorhabdus* species. Core genes clusters (GCs) were those found in all four genomes, and numbered 2364. Accessory GCs were those found in two or three genomes. BG5-BMMCB, BG5-VH1-XN45 and BMMCB-VH1-XN45 accessory genomes had 150, 297 and 154 GCs respectively. Strain-specific GCs were those found in one genome only, and BMMCB, BG5, XN45 and VH1 had 766, 377, 54 and 26 strain-specific GCs respectively. (b) Bar charts of known functions encoded by *Xenorhabdus* sp. strain BG5 (strain BG5) GCs. Commensurate with their lifestyle as endosymbionts of entomopathogenic nematodes, these bacteria encoded the following non-canonical core functions: antibiotic resistance, and biosynthesis of specialized metabolites and toxins. For XN45-VH1-BG5 accessory GCs, those that encoded the metabolism and transport of carbohydrates such as apiose, fuculose, tagatose, galactose and sorbose, and those that encoded biosynthesis of specialized metabolites such as antibiotics, polyketides, non-ribosomal peptides and siderophores were enriched. For BG5-BMMCB accessory GCs, those that encoded the biosynthesis of specialized metabolites were enriched. For BG5 strain-specific GCs, those that encoded biosynthesis of specialized metabolites and toxins such as type II, III and IV secretion system toxins were enriched among those with known protein functions.

A pangenome analysis of the clade containing strains XN45, VH1, BG5 and BMMCB, ‘the *X. griffiniae’* clade, was conducted. It had 15 411 genes that formed 4877 GCs. There were 2364 core GCs, of which 2231 were single-copy. Other groups included 766 strain BMMCB specific, 617 XN45-VH1 species specific, 377 strain BG5 specific, 297 BG5-VH1-XN45 accessory, 154 BMMCB-VH1-XN45 accessory, 150 BG5-BMMCB accessory, 54 strain XN45 specific, 26 strain VH1 specific, 14 XN45-BG5 accessory and five VH1-BMMCB accessory GCs (Fig. 3). From an analysis of gene gain and loss within this clade (Fig. S2), the species-specific genes, 79% of which encoded proteins with unknown functions, possibly resulted from a net acquisition of new genes [55].

To determine which encoded functions were enriched in the core, accessory and strain-specific genomes, single-copy GCs of each were annotated in PROKKA [43]. The functions and biological processes that the GCs encoded were then determined from the UniProt Knowledgebase [44] and COG [45] descriptions. In total, 3352 (68.4 %) GCs had known functions. The core genome had the largest percentage of these while strain-specific genomes had the smallest. Specifically, 80 % (1735/2182) of the core, 46 % (117/252) of BG5-XN45-VH1 accessory, 36 % (38/107) of BG5-BMMCB accessory, 29 % (31/108) of BMMCB-XN45-VH1 accessory, 23 % (75/327) of strain BG5 specific, 23 % (154/655) of strain BMMCB specific and 18 % (102/561) of *X. griffiniae* species specific GCs had known functions (Figs 3 and S1).

The most enriched functions in the core were those of housekeeping, such as translation, ribosome structure and biogenesis, amino acid transport and metabolism, carbohydrate transport and metabolism, and cell wall/membrane/envelope (Fig. 3). For the last, the Gram-negative nature of the bacteria [30] was demonstrated by the presence of genes encoding lipopolysaccharide (LPS) biosynthesis such as *lpt, rfa, lpx* operons, *lapA-B*, *msbA*, *waaA*, *galU*, *wbgU* and *yhjD. Xenorhabdus* are characterized as motile, peritrichously flagellated rods [30] and this was demonstrated by the presence of genes encoding flagellum biogenesis and motility such as *flgA*, *flgD, flgJ-L, flhA-D, fliC-T* and *fliZ*. Strains XN45, VH1 and BG5 exhibited swarming motility, and this was supported by the presence of the following core genes: *flgB-C*, *flgE-G* and *motA-B*. Other core genes included those encoding antibiotic resistance such as *acrABRZ, mdtABCK macAB fsr bsr* and *lmrA*. The biosynthesis of a few specialized metabolites was also a core function (Fig. 3), and this corroborated a pangenome analysis of genes encoding specialized metabolites from both *Xenorhabdus* and *Photorhabdus* bacteria [28].

*X. griffinae* genomes (XN45 and VH1) were enriched with a wide selection of genes that encoded carbohydrate metabolism and transport. This was demonstrated by enrichment of genes encoding metabolism and transport of apiose, fuculose, tagatose, galactose and sorbose in the XN45-VH1-BG5 accessory GCs, ribose, galactose, *myo*-inositol, d-malate and galactonate in the XN45-VH1-BMMCB GCs, and glucoside, glycolate and glycerate in the *X. griffiniae* species-specific GCs (Figs 3 and S1). Biosynthesis of specialized metabolites – such as non-ribosomal peptides, polyketides siderophores and antibiotics – was highly species dependent, as demonstrated by its enrichment in species-specific GCs. This was similarly observed with the production of type II, III and IV secretion system toxins. However, these enrichments were from a small fraction (21 %) of species-specific GCs; most genes specific to these three *Xenorhabdus* species encoded proteins with unknown functions.

### *Xenorhabdus* sp. BG5 genomic islands are flanked by transposase genes

**Fig. 4.**
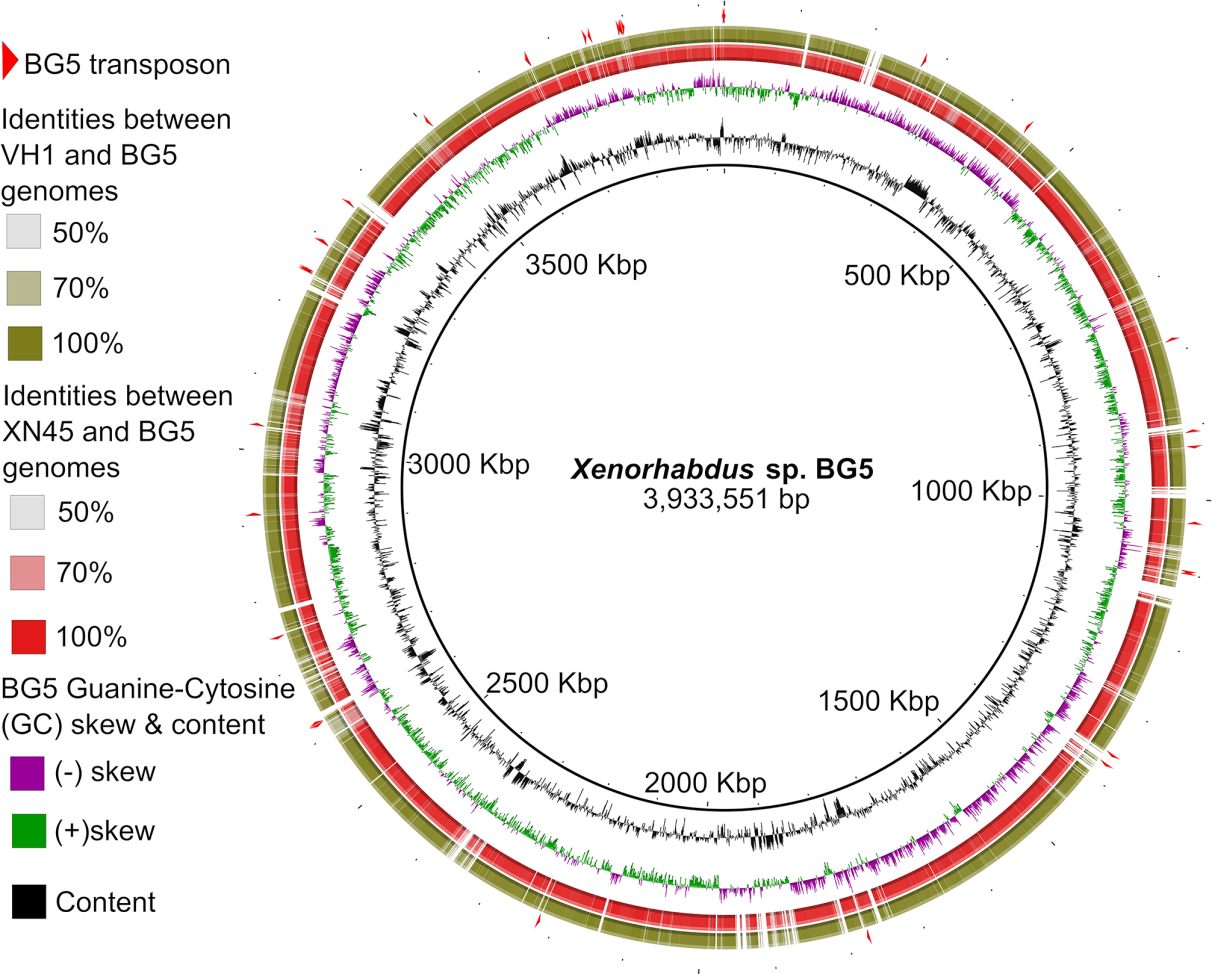
Genome visualizations of *Xenorhabdus* sp. BG5 when compared to *X. griffiniae* XN45 (red) and *X. griffiniae* VH1 (green). Genomic islands of *Xenorhabdus* sp. BG5 are denoted by white breaks in *X. griffiniae* genomes, and these represented cognate nucleotide sequences that were less than 50 % identical. Red triangles in the outermost ring denote loci of IS family transposase genes on the BG5 genome. All transposase genes that were not on contig edges flanked genomic islands. Genomic islands not associated with transposase genes are also shown. The visualization was created in Blast Ring Image Generator (BRIG).

To investigate whether genes specific to *Xenorhabdus* sp. BG5 formed genomic islands, its genome was compared to those of *X. griffiniae* XN45 and VH1. Regions of less than 50 % nucleotide similarity were determined and visualized in BRIG [47]. These were verified as genomic islands by genome alignments in Mauve. Most CDS in these genomic islands encoded hypothetical proteins. Genes encoding transposases in BG5 either flanked genomic islands/were the genomic island (Fig. 4). Insertion sequence (IS) elements predominated the type of transposases predicted to be encoded by these genes, as shown here: IS110 family transposase ISSfl8, IS3 family transposase ISKpn37, IS481 family transposase ISVvu4, IS5 family transposase ISSod6, IS630 family transposase ISPlu10, IS1 family transposase ISEhe5, IS1 family transposase ISPda1, IS110 family transposase ISPlu13, IS3 family transposase ISAlg, IS630 family transposase ISEc40 and IS982 family transposase ISNsp1 (workbook S1). IS elements contribute to genome reshuffling [56] and thus may be implicated in the creation of genomic islands in *Xenorhabdus* sp. BG5.

## Discussion

This study aimed to discover new *Xenorhabdus* strains because different species [2, 57] and even strains [3] from this genus have different antimicrobial production profiles. Soils of Western Kenya were selected as they had not hitherto been investigated for steinernematids, unlike those from Central Kenya [16, 21]. Soils were collected from Bungoma County and sites with the occurrence of steinernematids were clay soils on riverine land, corroborating previous studies on similar soils [58]. From Vihiga County, soils with the occurrence of steinernematids were red volcanic loam soils on cabbage cultivated land, corroborating previous studies on similar lands [21, 59]. From nematodes isolated from these soils, *Xenorhabdus* sp. strains VH1 and BG5 were isolated.

Previously we isolated *X. griffiniae* XN45 from *Steinernema* sp. scarpo which also originated from Kenyan soils. Using draft genome assemblies of strains XN45, VH1 and BG5 (Table 1) which were all of suitable quality for species delineation as per the standards of Chun *et al*. [23], the phylogenomic reconstruction of the genus (Fig. 1) demonstrated that these Kenyan strains formed a monophyletic group that could be enlarged to include strain BMMCB. This strain was previously designated as representing an *X. griffiniae* species. The draft assembly of XN45 was used for species delineation via analysis of orthoANI, DDH and G+C content thresholds for conspecific strains [24, 25, 51]. From these, strain BMMCB was identified as an undescribed species whereas strain VH1 was designated as *X. griffiniae* VH1. Strain BG5 was most closely related to XN45. However, ANI, dDDH and GGD values for the two were not consistent with those of conspecific strains. It was thus designated as an undescribed species of the genus *Xenorhabdus *. This demonstrated the importance of genome assemblies for accurate species delineation via overall genome relatedness indices, corroborating previous pivotal studies [23, 26, 60].

The open pangenome of the genus *Xenorhabdus* corroborated not only a larger pangenome analysis of 40 *Xenorhabdus* strains [54] but also the large number of *Xenorhabdus* strains –hosted by over 50 described *Steinernema* species [19] – that have yet to be isolated, identified and their genome sequences determined.

Using a pangenome analysis of the clade, the four closely related strains XN45, VH1, BG5 and BMMCB were further distinguished as representing three species based on the large numbers of species-specific genes – these were 377, 617 and 766 for *Xenorhabdus* sp. BG5, *X. griffiniae* and *Xenorhabdus* sp. BMMCB respectively. Conversely, strain VH1 was of the same species as XN45 as its genome only had 26 unique genes when compared to that of XN45. Similar pangenome analyses of other clades may elucidate a minimum number of strain-specific genes that delineate species in this genus. Notably, 79 % of the functions of proteins encoded by species-specific genes were unknown, compared to 20 % for proteins encoded by the core genome. It has long been established that most species-specific prokaryotic genes encode unknown functions [61]. Indeed, in 613 prokaryotic species, over 50 % of a subset of species-specific protein-coding genes encoded unknown functions [62]. These genes lead to speciation when they encode environmentally important traits [61]. For all three species, species-specific genes – only the subset that had known functions – were enriched for the biosynthesis of specialized metabolites. However, this enrichment was probably overestimated because genes encoding secondary metabolites of *Xenorhabdus* species are often long and clustered in genome loci that span thousands of base pairs, which leads to their frequent fragmentation in draft genomes, resulting in inflated counts [63]. *Xenorhabdus* sp. BG5 had genomic islands when its genome was compared to those of *X. griffiniae* strains. Some of these islands were flanked by genes encoding transposases, the vast majority of which were IS elements. IS elements are known to contribute to genome reshuffling [56] suggesting that IS transposases contributed to the creation of genomic islands in *Xenorhabdus* sp. BG5. However, the majority of the islands were not associated with genes encoding transposases, implicating other factors such as phages, as drivers of these differences. In conclusion, two *Xenorhabdus* bacteria isolated from steinernematids from soils in Western Kenya were identified as a novel species and strain. Within the *X. griffiniae* clade, most species-specific genes encoded unknown functions. These genomes, species delineations and genome analyses are useful for *in silico*-based discovery of antimicrobials from the genus *Xenorhabdus *.

## Supplementary Data

Supplementary material 1
