# Supplementary material for: Draft genomes, phylogenomic reconstruction and comparative genome analysis of three Xenorhabdus strains isolated from soil-dwelling nematodes in Kenya
Source: Access Microbiol. 2023 May 22;5(5):acmi000531.v4. doi: 10.1099/acmi.0.000531.v4 (PMC10267655; doi:10.1099/acmi.0.000531.v4)
Supplement: Supplementary material 1 [file acmi-5-531.v4-s001.pdf]

# Draft genomes, phylogenomic reconstruction and comparative genome analysis of three *Xenorhabdus* strains isolated from soil dwelling nematodes in Kenya

Ryan Musumba Awori[1,2*], Charles N. Waturu[3], Sacha J. Pidot[4], Nelson O. Amugune[5], Helge B. Bode[1,6,7,8]

[1]Department of Biosciences, Goethe University Frankfurt, 60438 Frankfurt am Main, Germany.

[2]Elakistos Biosciences, P. O. Box 19301-00100, Nairobi, Kenya.

[3] Horticultural Research Institute, Kenya Agricultural and Livestock Research Organisation, P. O. Box 220 Thika.

[4]Department of Microbiology and Immunology at the Doherty Institute, University of Melbourne, Australia.

[5]Department of Biology, University of Nairobi, P. O. Box 30197-00100, Nairobi, Kenya

[6]Department of Natural Products in Organismic Interactions, Max Planck Institute for Terrestrial Microbiology, 35043 Marburg, Germany.

[7]Chemical Biology, Department of Chemistry, Phillips University Marburg, 35043 Marburg, Germany.

[8]Senckenberg Gesellschaft für Naturforschung, 60325 Frankfurt am Main, Germany

**Supplementary Figures and Tables**

Author for correspondence: Ryan Musumba Awori, Elakistos Biosciences, P. O. Box 19301-00100, Nairobi, Kenya, ryan-musumba.awori@elakistosbiosciences.com

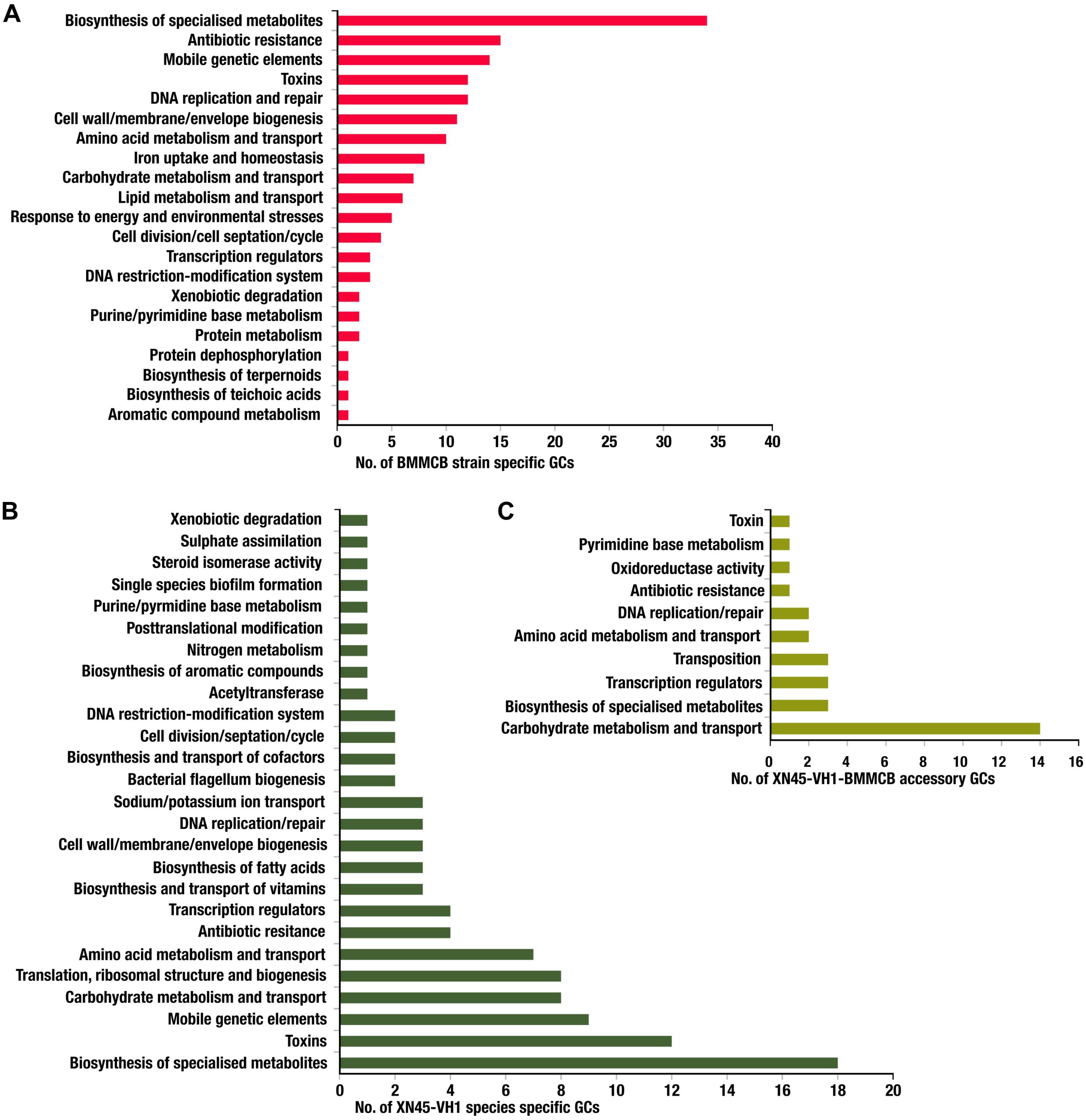


**Supplementary Figure S1.** A) Bar chart of known protein functions encoded by single copy genes of the *Xenorhabdus* sp. BMMCB strain specific gene clusters (GCs). GCs that encoded biosynthesis of specialised metabolites such as non-ribosomal peptides, polyketides, antibiotics, siderophores, and antibiotic resistance such as efflux pumps, MFS transporters, and beta-lactamases were enriched among those with known protein functions. B) Bar chart of known protein functions encoded by single copy GCs of the *Xenorhabdus griffiniae* specific genome. GCs that encoded biosynthesis of specialised metabolites were enriched. C) Bar chart of known functions encoded by single copy GCs of the *Xenorhabdus*

sp. BMMCB-*X. griffiniae* accessory genome. GCs that encoded the metabolism and transport of carbohydrates such as ribose, galactose, myo-inositol, D-malate, and galactonate were enriched among those with known protein functions.

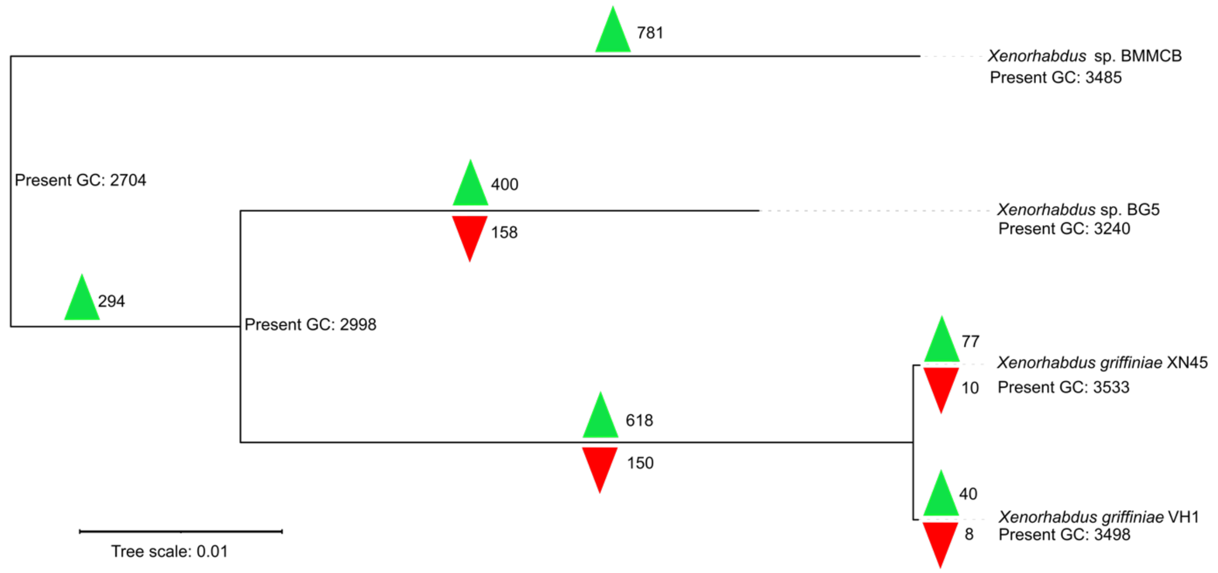


**Supplementary Figure S2**. Evolution of the number of gene clusters in the *Xenorhabdus griffiniae* clade. GC denotes total gene clusters present in either an extant or extinct genome. Green and red triangles denote gene gains and losses respectively.

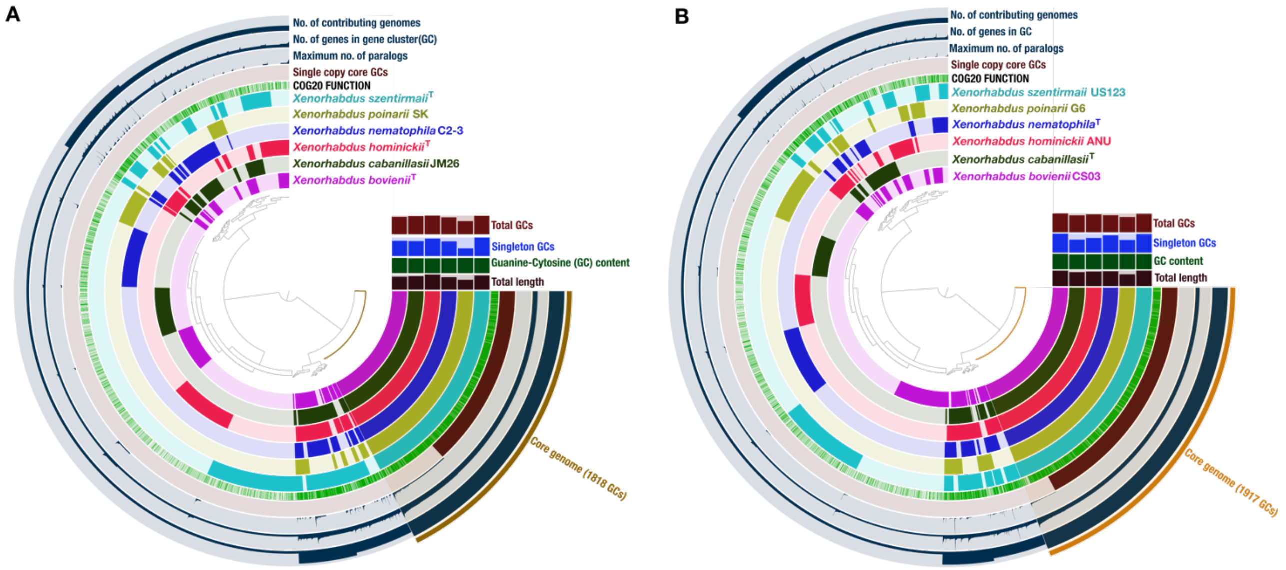


**Supplementary Figure S3**. Comparison of core genomes from two pangenomes of similar *Xenorhabdus* species created from draft and complete (composed of less than two contigs) genomes. For the pangenome made from A) draft genomes, the number of core, and single copy core GCs were 1818 and 1365 respectively. For the pangenome made from B) complete genomes, the number of core and single copy core gene clusters (GCs) were 1917 and 1728 respectively. Thus, draft genomes underestimated the number of core and single copy core GCs by 5% and 21% respectively.

**Supplementary Table S1.** *Steinernema* isolates from locations in Sub-Saharan Africa.

| Isolate | Location | Xenorhabdus symbiont | Reference |
|---|---|---|---|
| *Steinernema pwaniensis* | Mwalusembe, Tanzania | No published isolate | Půža *et al.,* 2017 |
| *Steinernema* sp. Thui | Akoutaossé, Benin | *X. indica* | Godjo *et al.,* 2018 |
| *S. cameroonense* | Obala, Cameroon | *Xenorhabdus* sp. A71 | Kanga *et al.,* 2012; Kanga *et al.,* 2014 |
| *S. nyetense* | Nyété, Cameroon | No published isolate | Kanga *et al.,* 2012 |
| *S. ethiopiense* | Mendi area, Ethiopia | No published isolate | Tamiru *et al.,* 2012 |
| *S. yirgalemense* | Yirgalem, Ethiopia | *X. indica* | Ferreira *et al.,* 2016; Nguyen *et al.,* 2004 |
| *S. nguyeni* | Clanwilliam S. Africa | *X. bovienii* | Antoinette P. Malan *et al.,* 2016; Dreyer *et al.,* 2017 |
| *S. tophus* | Clanwilliam S. Africa | No published isolate | Cimen *et al.,* 2014 |
| *S. sacchari* | Gingindlovu, S. Africa | *X. khoisanae* | Dreyer *et al.,* 2017; Nthenga *et al.,* 2014 |
| *S. fabii* | Piet Retief, S. Africa | No published isolate | Abate *et al.,* 2016 |
| *S. bertusi* | Port Edward, S. Africa | No published isolate | Katumanyane *et al.,* 2020 |
| *S. citrae* | Piketberg, S. Africa | No published isolate | Nomakholwa *et al.,* 2011 |
| *S. innovationi* | Free State, S. Africa | No published isolate | Çimen *et al.,* 2015 |
| *S. jeffreyense* | Jefferson Bay, S. Africa | *X. khoisanae* | A.P. Malan *et al.,* 2016; Dreyer *et al.,* 2017 |
| *S. beitlechemi* | Bethlehem, S. Africa | *X. khoisanae* | Cimen *et al.,* 2016 |
| *S. khoisanae* | Villiersdorp, S. Africa | *X. khoisanae* | Ferreira *et al.,* 2013; Malan *et al.,* 2006 |
| *Steinernema* sp. WS9 | Fridenheim, S. Africa | *X. griffiniae* | Jonike Dreyer *et al.,* 2018 |
| *S. australe* TEL | Walkerville, South Africa | No published isolate | Lephoto & Gray 2019 |
| *Steinernema* sp. HBG28 | Guateng, S. Africa | *X. khoisanae* | Naidoo *et al.,* 2015 |
| *Steinernema* sp. LAOS | S. Africa | *Xenorhabdus* sp. strain GDc328 | Soobramoney *et al.,* 2015 |
| *Steinernema* sp. BMMCB | Brits, S. Africa | *Xenorhabdus* sp. BMMCB | Mothupi *et al.,* 2015 |

**Supplementary Table S2**. Soil samples names and their geographic regions of collection. Steinernematids were isolated from those in bold

| Sample name | Latitude | Longitude | Altitude(m) | Area description |
|---|---|---|---|---|
| TN1 | 0.01971 | 35.44755 | 2485.7 | Virgin forest |
| TN3 | 0.01968 | 35.44765 | 2486.7 | Virgin forest |
| TN4 | 0.01968 | 35.44765 | 2486.7 | Virgin forest |
| TN5 | 0.01926 | 35.44772 | 2478.7 | Virgin forest |
| TN6 | 0.01908 | 35.44796 | 2478.7 | Virgin forest |

| | | | | |
|---|---|---|---|---|
| TN7 | 0.01908 | 35.44804 | 2451.7 | Virgin forest |
| TN8 | 0.01673 | 35.4545 | 2595.7 | Cultivated land with minimum disturbance |
| TN9 | 0.01662 | 35.45479 | 2613.7 | Cultivated land with minimum disturbance |
| TN10 | 0.01647 | 35.45539 | 2629.7 | Cultivated land with minimum disturbance |
| TN11 | 0.02258 | 35.46348 | 2773.6 | Virgin forest |
| TN12 | 0.02287 | 35.46344 | 2744.0 | Virgin forest |
| TN13 | 0.02223 | 35.46351 | 2694.0 | Virgin forest |
| TN14 | 0.02223 | 35.46341 | 2672.0 | Virgin forest |
| ND1 | 0.1053 | 35.19019 | 2045.0 | Near tea cultivated land |
| ND2 | 0.10535 | 35.19028 | 2062.0 | Tea cultivated land |
| ND3 | 0.10498 | 35.19019 | 2047.0 | Near tea cultivated land |
| ND4 | 0.10355 | 35.19836 | 2017.0 | Eucalyptus cultivated land |
| ND5 | 0.10309 | 35.19858 | 1989.0 | Eucalyptus cultivated land |
| ND6 | 0.10207 | 35.19084 | 2064.0 | Tea cultivated land |
| ND7 | 0.10219 | 35.19069 | 2055.0 | Tea cultivated land-1990 tea bush |
| ND8 | 0.0926 | 35.19466 | 1967.0 | Eucalyptus cultivated land |
| ND9 | 0.0884 | 35.19389 | 1934.0 | Riverine vegetation |
| ND10 | 0.08779 | 35.19403 | 1930.0 | Riverine vegetation |
| ND11 | 0.08725 | 35.21023 | 2019.0 | Tea cultivated land- 1996 tea bush |
| ND12 | 0.08631 | 35.21016 | 2010.6 | Indigenous trees cultivated land |
| ND13 | 0.0862 | 35.21019 | 1996.0 | Indigenous trees cultivated land |
| ND14 | 0.08594 | 35.21032 | 1981.6 | Riverine vegetation |
| KR1 | -0.1957 | 35.28344 | 1422.7 | Fallow land |
| KR2 | -0.19564 | 35.2839 | 1420.0 | Fallow land |
| KR3 | -0.21374 | 35.31923 | 1429.4 | Riverine vegetation |
| KR4 | -0.21363 | 35.31924 | 1491.4 | Riverine vegetation |
| KS1 | -0.12548 | 34.7447 | 1143.0 | River Wigia Inlet |
| KS2 | -0.12547 | 34.7464 | 1143.0 | River Wigia Inlet swamp area |
| KS4 | -0.12565 | 34.74166 | 1117.0 | Hippo Point |
| KS5 | -0.12537 | 34.74238 | 1117.0 | Hippo Point-River inlet swamp area |
| KS6 | -0.12541 | 34.74238 | 1117.0 | Hippo Point |
| KS7 | -0.13592 | 34.74103 | 1133.0 | Swamp Land |
| KS8 | -0.08592 | 34.74627 | 1130.0 | Lake Victoria shoreline |
| KS9 | -0.08596 | 34.74644 | 1130.0 | Lake Victoria shoreline |
| KS10 | -0.08596 | 34.74654 | 1130.0 | Lake Victoria shoreline |
| KS11 | -0.087818 | 34.74231 | 1139.0 | Lake Victoria shoreline |
| KS12 | -0.08756 | 34.7432 | 1139.0 | Lake Victoria shoreline |
| KS13 | -0.08761 | 34.74207 | 1139.0 | Lake Victoria shoreline |
| KS14 | -0.08774 | 34.74140 | 1140.0 | Lake Victoria shoreline |
| GS1 | 0.0577 | 34.76411 | 1680.0 | Cultivated land |
| GS2 | 0.05824 | 34.76421 | 1680.0 | Cultivated land-Maize |
| GS3 | 0.05857 | 34.76486 | 1668.0 | Eucalyptus cultivated land |
| GS4 | 0.05895 | 34.76526 | 1634.6 | Riverine vegetation |
| GS5 | 0.05913 | 34.76493 | 1656.3 | Man-made pond banks |
| GS6 | 0.05623 | 34.77107 | 1719.6 | Virgin land |
| **VH1** | **0.06293** | **34.72903** | **1624.0** | **Cultivated land-cabbage** |
| VH2 | 0.0629 | 34.72911 | 1624.0 | Cultivated land-cabbage |
| VH3 | 0.06644 | 34.7316 | 1592.0 | Virgin forest |

| | | | | |
|---|---|---|---|---|
| VH4 | 0.06637 | 34.73195 | 1592.0 | Virgin forest |
| ST1 | 0.12841 | 34.85427 | 1660.0 | Virgin tropical rain forest |
| ST2 | 0.12972 | 34.8464 | 1657.0 | Virgin tropical rain forest |
| ST3 | 0.12978 | 34.84423 | 1657.0 | Virgin tropical rain forest |
| ST4 | 0.12968 | 34.84413 | 1610.0 | Virgin tropical rain forest |
| KG1 | 0.17356 | 34.89599 | 1627.0 | Virgin tropical rain forest |
| KG2 | 0.17382 | 34.8959 | 1627.0 | Virgin tropical rain forest |
| KG3 | 0.15393 | 34.8935 | 1618.0 | Kakabuti Riverbed |
| KG4 | 0.15375 | 34.89375 | 1618.0 | Virgin tropical rain forest |
| BY1 | 0.48037 | 34.40839 | 1240.0 | Virgin riverbank land |
| **BG5** | **0.48044** | **34.40836** | **1239.0** | **Virgin riverbank land** |
| BY2 | 0.48035 | 34.40849 | 1239.0 | Virgin riverbank land |
| BY3 | 0.48043 | 34.40851 | 1239.0 | Virgin riverbank land |
| BY4 | 0.48051 | 34.40845 | 1239.0 | Virgin riverbank land |
| BY5 | 0.48046 | 34.40828 | 1239.0 | Virgin riverbank land |
| BY6 | 0.48419 | 34.373222 | 1239.0 | Cultivated land- Maize |
| BY7 | 0.48466 | 34.37534 | 1230.0 | Cultivated land- cassava |
| BY8 | 0.4857 | 34.3763 | 1230.0 | Fallow land |
| BY9 | 0.48584 | 34.37654 | 1215.0 | Riverine vegetation |
| BY10 | 0.48575 | 34.37653 | 1229.0 | Riverine vegetation |
| BY11 | 0.48937 | 34.37311 | 1229.0 | Riverine vegetation |
| EL1 | 0.882906 | 34.731095 | 2149.0 | Fallow land |
| EL2 | 0.882906 | 34.731095 | 2149.0 | Fallow land |

**Supplementary Table S3.** Accessions numbers, strains and predicted gene counts of genomes used in phylogenomic and pangenomic analyses.

| Genbank number of master- record for drafts/complete genome | Strain name | For pangenome analyses | | |
|---|---|---|---|---|
| | | Number of genes called | Number of excluded partial gene calls | Number of gene calls used |
| JACWFC000000000.1 | *Xenorhabdus griffiniae* XN45 | 3957 | 274 | 3683 |
| JADEUF000000000.1 | *X. griffinae* VH1 | 3853 | 196 | 3657 |
| JADEUG000000000.1 | *Xenorhabdus* sp. BG5 | 3575 | 96 | 3479 |
| MUBK01000000 | *X. beddingii* DSM 4764 | 3735 | 219 | 3516 |
| JANAIF000000000.1 | *X. bovienii* T228 | 4337 | 480 | 3857 |
| NIBS00000000.1 | *X. budapestensis* DSM 16342 | 3807 | 168 | 3639 |
| NZ_QTUB01000001 | *X. cabanillasii* DSMZ 19705 | 3822 | 1 | 3821 |
| GCA_000968195.1 | *X. doucetiae* FRM16 | 3653 | 1 | 3652 |
| MKGQ00000000.1 | *X. eapokensis* DL20 | 3895 | 175 | 3720 |
| NIBT00000000.1 | *X. ehlersii* DSM 16337 | 3978 | 96 | 3882 |
| NJAI00000000.1 | *X. hominickii* DSM 17903 | 4955 | 152 | 4803 |
| NIBU00000000.1 | *X. innexi* DSM 16336 | 4262 | 328 | 3934 |
| NJAK00000000.1 | *X. ishibashii* DSM 22670 | 3557 | 4 | 3553 |
| FOVO00000000.1 | *X. japonica* DSM 16522 | 3229 | 51 | 3178 |
| LFCV00000000.1 | *X. khoisanae* MCB | 4182 | 197 | 3985 |
| FPBJ00000000.1 | *X. koppenhoeferi* DSM 18168 | 2771 | 37 | 2733 |

| | | | | |
|---|---|---|---|---|
| NJCX00000000.1 | *X. kozodoii* DSM 17907 | 3733 | 133 | 3600 |
| JACOII000000000.1 | *X. lircayensis* VLS | 3909 | 79 | 3830 |
| NITY00000000.1 | *X. mauleonii* DSM 17908 | 4446 | 118 | 4328 |
| NITZ00000000.1 | *X. miraniensis* DSM 17902 | 4357 | 139 | 4218 |
| GCA_000953355.1 | *X. nematophila* AN6/1 | 4127 | 0 | 4127 |
| FO704551 | *X. poinarii* G6 | 3357 | 0 | 3357 |
| NJAJ00000000.1 | *X. stockiae* DSM 17904 | 4164 | 194 | 3970 |
| NIBV00000000.1 | *X. szentirmaii* DSM16338 | 4257 | 80 | 4177 |
| MKGR00000000.1 | *X. thuongxuanensis* 30TX1 | 3560 | 101 | 3459 |
| MUBJ00000000.1 | *X. vietnamensis* DSM 22392 | 3998 | 134 | 3864 |
| LDNM00000000.1 | *Xenorhabdus* sp. BMMCB | 4024 | 214 | 3810 |

**References**


Abate BA et al. 2016. *Steinernema fabii* n. sp.(Rhabditida: Steinernematidae), a new entomopathogenic nematode from South Africa. Nematology. 18:235–255.

Cimen H et al. 2016. *Steinernema beitlechemi* n. sp., a new entomopathogenic nematode (Nematoda: Steinernematidae) from South Africa. Nematology. 18:439–453.

Çimen H, Lee M-M, Hatting J, Hazir S, Stock SP. 2015. *Steinernema innovationi* n. sp. (*Panagrolaimomorpha: Steinernematidae*), a new entomopathogenic nematode species from South Africa. Journal of Helminthology. 89:415–427. doi: 10.1017/S0022149X14000182.

Cimen H, Lee MM, Hatting J, Hazir S, Stock SP. 2014. *Steinernema tophus* sp. n.(Nematoda: *Steinernematidae*), a new entomopathogenic nematode from South Africa. Zootaxa. 3821:337–353.

Dreyer J, Malan AP, Dicks LMT. 2018. First report of a symbiotic relationship between *Xenorhabdus griffiniae* and an unknown *Steinernema* from South Africa. Archives of Microbiology. 200:349–353. doi: 10.1007/s00203-017-1452-4.

Dreyer J, Malan AP, Dicks LMT. 2017. Three Novel *Xenorhabdus–Steinernema* Associations and Evidence of Strains of *X. khoisanae* Switching Between Different Clades. Current Microbiology. 74:938–942. doi: 10.1007/s00284-017-1266-2.

Ferreira T et al. 2013. Description of *Xenorhabdus khoisanae* sp. nov., the symbiont of the entomopathogenic nematode *Steinernema khoisanae*. International Journal of Systematic and Evolutionary Microbiology. 63:3220–3224.

Ferreira T et al. 2016. First report of the symbiotic bacterium *Xenorhabdus indica* associated with the entomopathogenic nematode *Steinernema yirgalemense*. Journal of Helminthology. 90:108–112. doi: 10.1017/S0022149X14000583.

Godjo A, Afouda L, Baimey H, Decraemer W, Willems A. 2018. Molecular diversity of *Photorhabdus* and *Xenorhabdus* bacteria, symbionts of *Heterorhabditis* and *Steinernema* nematodes retrieved from soil in Benin. Archives of Microbiology. 200:589–601.

Kanga FN et al. 2012. Two new species of *Steinernema* Travassos, 1927 from the humid forest of southern Cameroon. Russian Journal of Nematology. 20.

Kanga FN, Ivanova ES, Shepeleva NS, Spiridonov SE. 2014. Additional data on *Steinernema cameroonense* Ngo Kanga, Phap Quang Trinh, Wayenberge, Spiridonov, Hauser & Moens, 2012. Russian Journal of Nematology. 22.

Katumanyane A, Malan AP, Tiedt LR, Hurley BP. 2020. *Steinernema bertusi* n. sp.(*Rhabditida: Steinernematidae*), a new entomopathogenic nematode from South Africa. Nematology. 22:343–360.

Lephoto TE, Gray VM. 2019. First report of the isolation of entomopathogenic nematode *Steinernema australe* (Rhabditida: Steinernematidae) from South Africa. South African Journal of Science. 115:1–5.

Malan A, Gozel U, Nguyen K. 2006. *Steinernema khoisanae* n. sp.(*Rhabditida: Steinernematidae*), a new entomopathogenic nematode from South Africa. Nematology. 8:157–175.

Malan A.P., Knoetze R, Tiedt LR. 2016. *Steinernema jeffreyense* n. sp. (Rhabditida: *Steinernematidae*), a new entomopathogenic nematode from South Africa. Journal of Helminthology. 90:262–278. doi: 10.1017/S0022149X15000097.

Malan Antoinette P., Knoetze R, Tiedt LR. 2016. *Steinernema nguyeni* n. sp.(Rhabditida: *Steinernematidae*), a new entomopathogenic nematode from South Africa. Nematology. 18:571–590.

Mothupi B, Featherston J, Gray V. 2015. Draft Whole-Genome Sequence and Annotation of *Xenorhabdus griffiniae* Strain BMMCB Associated with the South African Entomopathogenic Nematode *Steinernema khoisanae* Strain BMMCB. Genome Announcements. 3. doi: 10.1128/genomeA.00785-15.

Naidoo S, Featherston J, Gray VM. 2015. Draft Whole-Genome Sequence and Annotation of the Entomopathogenic Bacterium *Xenorhabdus khoisanae* Strain MCB. Genome Announcements. 3. doi: 10.1128/genomea.00872-15.

Nguyen KB, Tesfamariam M, Gozel U, Gaugler R, Adams BJ. 2004. *Steinernema yirgalemense* n. sp. (*Rhabditida: Steinernematidae*) from Ethiopia. Nematology. 6:839–856. doi: 10.1163/1568541044038605.

Nthenga I, Knoetze R, Berry S, Tiedt LR, Malan AP. 2014. *Steinernema sacchari* n. sp.(*Rhabditida: Steinernematidae*), a new entomopathogenic nematode from South Africa. Nematology. 16:475–494.

Půža V, Nermut J, Mráček Z, Gengler S, Haukeland S. 2017. *Steinernema pwaniensis* n. sp., a new entomopathogenic nematode (*Nematoda: Steinernematidae*) from Tanzania. Journal of Helminthology. 91:20–34. doi: 10.1017/S0022149X15001157.

Soobramoney L-AO, Featherston J, Gray VM. 2015. Draft Whole-Genome Sequence of *Xenorhabdus* sp. Strain GDc328, Isolated from the Indigenous South African Nematode Host *Steinernema khoisanae*. Genome Announcements. 3:e01239-15. doi: 10.1128/genomeA.01239-15.

Tamiru T et al. 2012. *Steinernema ethiopiense* sp. n.(*Rhabditida: Steinernematidae*), a new entomopathogenic nematode from Ethiopia. Nematology. 14:741–757.
